# NLRP3 Localizes to the Tubular Epithelium in Human Kidney and Correlates With Outcome in IgA Nephropathy

**DOI:** 10.1038/srep24667

**Published:** 2016-04-20

**Authors:** Justin Chun, Hyunjae Chung, Xiangyu Wang, Rebecca Barry, Zohreh Mohammad Taheri, Jaye M. Platnich, Sofia B. Ahmed, Kiril Trpkov, Brenda Hemmelgarn, Hallgrimur Benediktsson, Matthew T. James, Daniel A. Muruve

**Affiliations:** 1 Department of Medicine, Snyder Institute for Chronic Diseases, University of Calgary, Calgary, Alberta, Canada; 2 Department of Pathology and Laboratory Medicine, Snyder Institute for Chronic Diseases, University of Calgary, Calgary, Alberta, Canada

## Abstract

Nod-like receptor pyrin domain-containing-3 (NLRP3) has been implicated in the pathogenesis of experimental renal injury, yet its characterization in human kidney disease remains largely unexplored. NLRP3 expression was evaluated in human kidney biopsies, primary renal tubular cells (HPTC) and correlated to disease outcomes in patients with IgA nephropathy (IgAN). NLRP3 localized to renal tubules in normal human kidney tissue and to mitochondria within HPTC by immunohistochemistry and immunofluorescence microscopy. Compared to control kidneys, NLRP3 gene expression was increased in biopsies of patients with IgAN. While NLRP3 expression in IgAN was detected in glomeruli, it remained largely confined to the tubular epithelial compartment. *In vitro* NLRP3 mRNA and protein expression were transiently induced in HPTC by TGF-β1 but subsequently diminished over time as cells lost their epithelial phenotype in a process regulated by transcription and ubiquitin-mediated degradation. Consistent with the *in vitro* data, low NLRP3 mRNA expression in kidney biopsies was associated with a linear trend of higher risk of composite endpoint of doubling serum creatinine and end stage renal disease in patients with IgAN. Taken together, these data show that NLRP3 is primarily a kidney tubule-expressed protein that decreases in abundance in progressive IgAN.

NLRP3 (NOD-like receptor, pyrin domain-containing 3) is a member of the NOD-like receptor (NLR) of innate immune genes. NLRP3 is best known for its role as a component of the inflammasome, which is a multi-protein caspase activating platform that regulates a variety of host defense pathways in response to pathogen or damage-associated molecular patterns (PAMPs or DAMPs, respectively). The NLRP3 inflammasome regulates caspase-1 activation which in turn regulates the maturation and secretion of pro-inflammatory cytokines such as IL-1β and IL-18^1^,^2^. There is growing evidence for inflammasome-independent or non-canonical roles for NLRP3 in the kidney tubular epithelial injury and fibrosis^3^,^4^,^5^,^6^,^7^. Given these properties, NLRP3 not surprisingly has been implicated in the pathogenesis of numerous kidney diseases at the experimental level including ischemia/reperfusion injury, unilateral ureteric obstruction, diabetic nephropathy, calcium oxalate-induced renal injury, diet-induced nephropathy and hyperhomocysteinemia^3^,^6^,^8^,^9^,^10^,^11^,^12^,^13^. However, despite the increasing number of reports describing a role for NLRP3 in animal renal injury models, the characterization of NLRP3 in the context of human kidney diseases remains largely unexplored.

IgA nephropathy (IgAN) is the most common type of primary glomerulonephritis in the world^14^. Although previously thought to have a relatively benign course, it is now known that 20–40% of patients with IgAN progress to end stage renal disease (ESRD) within 20 years^15^,^16^. IgAN is characterized by galactose-deficient IgA1 immune complex deposition in the glomerular mesangium that leads to activation of the complement cascade and other immunologic processes stimulating cell proliferation and secretion of growth factors, proinflammatory and profibrotic cytokines^17^,^18^. Glomerular inflammation leads to injury of podocytes and proximal tubular epithelial cells causing a sequelae of glomerulosclerosis, tubular injury/atrophy, and interstitial fibrosis. The mechanisms that initiate renal tubular injury downstream of glomerular inflammation remain poorly understood but tubular injury and interstitial fibrosis are critical to IgAN progression and remain the strongest pathologic predictors of disease outcome^19^. Given the current understanding of NLRP3 in the regulation of inflammation, tubular epithelial cell injury and fibrosis, the possibility exists that NLRP3 may be associated with the progressive chronic kidney disease induced by IgAN.

While NLRP3 is well known to be expressed in macrophages, its biology in kidney disease is believed to be largely non-canonical and dependent on non-hematopoietic cellular compartments^5^,^6^. Studies have demonstrated NLRP3 expression in tubular epithelial cells as well as podocytes that plays an important role in experimental disease pathogenesis^3^,^7^,^10^,^20^,^21^. Collectively, these data suggest that the biology of the NLRP3 in the kidney may differ from the canonical inflammasome pathway described in macrophages and other non-renal disease models that rely primarily caspase-1 activation and cytokine maturation^22^. Despite these observations, the cellular localization and characterization of NLRP3 in the human kidney or a temporal relationship to human kidney disease has yet to be confirmed. We previously demonstrated increased NLRP3 mRNA in kidney biopsies from a variety of nondiabetic kidney diseases including IgAN^6^. In this extension of our prior work, we employed human nephrectomy samples, kidney disease biopsies and primary tubular epithelial cells to characterize NLRP3 in the context of the human kidney and IgAN, a common chronic human kidney disease.

## Results

### NLRP3 localizes primarily to the tubular epithelium in the human kidney

**Figure 1:**
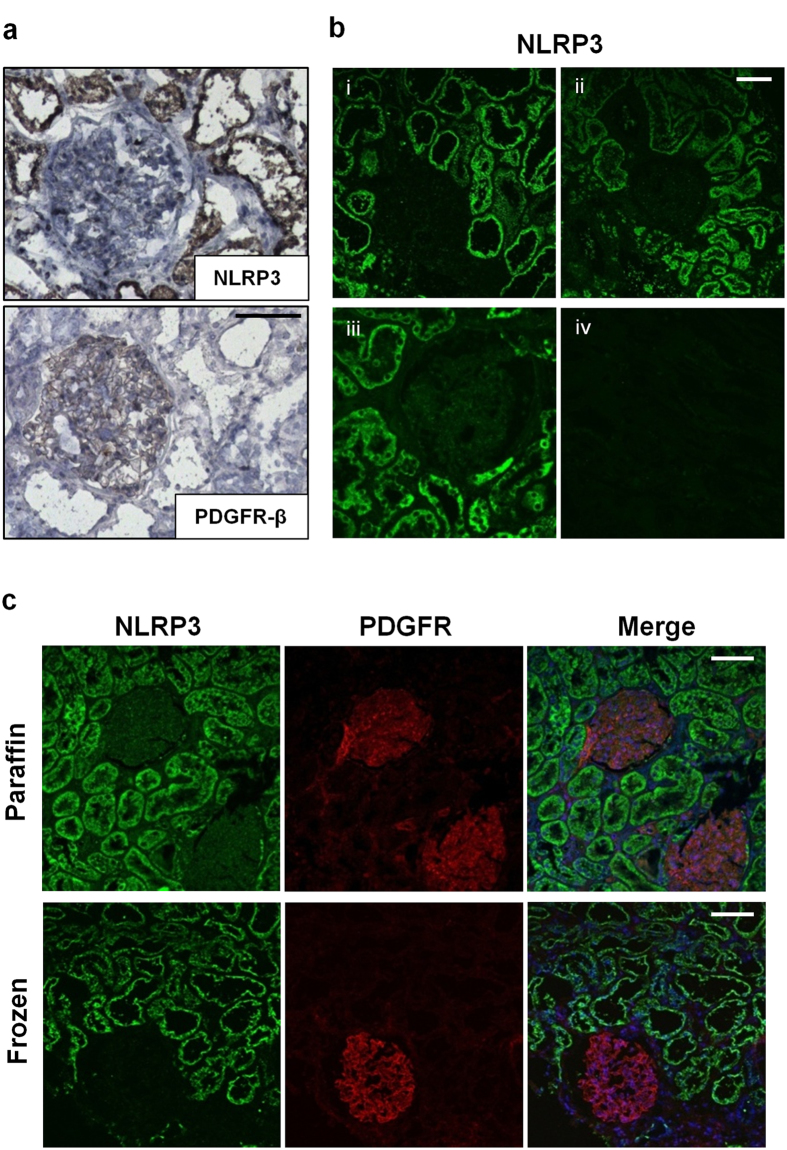
NLRP3 expression in normal human kidney. (**a**) Immunoperoxidase staining in frozen sections of normal kidney tissue (obtained from a representative human nephrectomy sample) using mouse monoclonal NLRP3 or rabbit polyclonal platelet derived growth factor receptor-β (PDGFR-β) antibodies. Scale bar 100 μm. (**b**) Indirect immunofluorescence for NLRP3 (Cryo2) in (i) frozen unfixed, (ii) methanol-acetone fixed or (iii) paraffin embedded normal kidney tissue. An antibody to NLRP3 (Sigma) was unable to detect NLRP3 in paraffin embedded samples (iv). Scale bar represents 50 μm. (**c**) Immunofluorescence using dual-labeling confocal microscopy probing for NLRP3 (green), PDGFR-β (red) and nuclei (DAPI: blue, in merged images) in paraffin embedded or frozen sections of normal kidney tissue. Scale bar represents 100 μm. Images are representative of experiments performed at least 3 independent times.

NLRP3 localization in the human kidney has been inconclusive with reported localization at podocytes and tubular epithelial cells^7^,^10^,^20^,^21^. To clarify NLRP3 localization in the human kidney, cryosections and paraffin embedded sections of histologically normal tissue obtained from human kidney nephrectomies were stained with immunoperoxidase or processed for indirect immunofluorescence (IF) and confocal microscopy. In normal kidney tissue stained with immunoperoxidase, NLRP3 localized primarily to tubules whereas platelet derived growth factor receptor-beta (PDGFR-β), a well-established marker of mesangial cells^23^,^24^, localized to glomeruli as expected (Fig. 1a). NLRP3 localization to tubules was verified in cryosections and paraffin embedded sections by IF (Fig. 1b,c). NLRP3 (Cryo-2; Adipogen) consistently and specifically detected NLRP3 at tubules in frozen or paraffin sections but an antibody to NLRP3 (Sigma) was unable to detect NLRP3 in paraffin embedded samples. Isotype controls were negative in all cases (Supplementary Fig. 1). Taken together, these results suggest that NLRP3 primarily localizes to the tubular epithelium in normal human kidney with virtually no glomerular localization.

**Figure 2:**
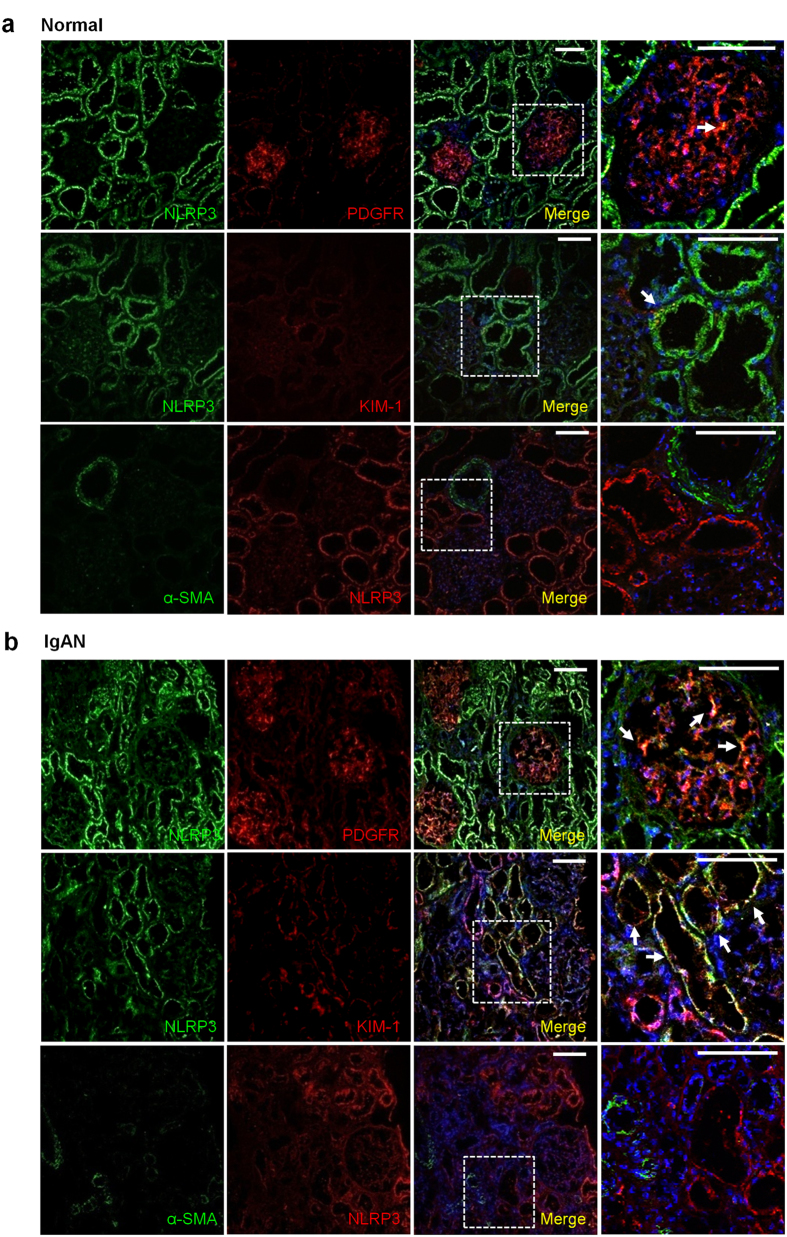
NLRP3 expression in human IgAN. Immunofluorescence confocal microscopy in frozen sections of normal kidney tissue (obtained from a representative human nephrectomy sample) (**a**) or a representative kidney biopsy from a patient with IgAN (**b**). Dual labeling probing for NLRP3 (green) and PDGFR-β (red) or kidney injury molecule-1 (KIM-1, red); NLRP3 (red) with alpha smooth muscle actin (α-SMA, green). Nuclear stain using DAPI (blue) is shown in merged images. Magnified views of the indicated regions outlined from the merged images. Scale bars 100 μm. Arrows point to areas of yellow indicating colocalization of green and red channels. Images are representative of experiments performed at least 3 independent times.

We have previously identified significant NLRP3 mRNA expression in the kidneys of a small number of patients with IgAN^6^. Thus, immunostaining experiments were performed in biopsies from patients with IgAN to further characterize NLRP3 in this cohort. Similar to normal kidney tissue, NLRP3 once again localized primarily to the tubular epithelium in patients with IgAN (Fig. 2a,b). Not surprisingly, with renal injury and disease, tissue from patients with IgAN also demonstrated increased KIM-1 expression, a tubule-restricted activation marker that colocalized with NLRP3 (Fig. 2b). In glomeruli, NLRP3 expression became detectable in IgAN and colocalized to PDGFR-β positive areas (Fig. 2b). NLRP3 staining was absent in fibrotic areas and did not colocalize with α-SMA in the interstitium ruling out NLRP3 expression in myofibroblasts. These results show that in IgAN, NLRP3 remains predominately expressed in tubules with lesser expression at glomeruli.

### NLRP3 localizes to mitochondria and is associated with the epithelial phenotype

**Figure 3:**
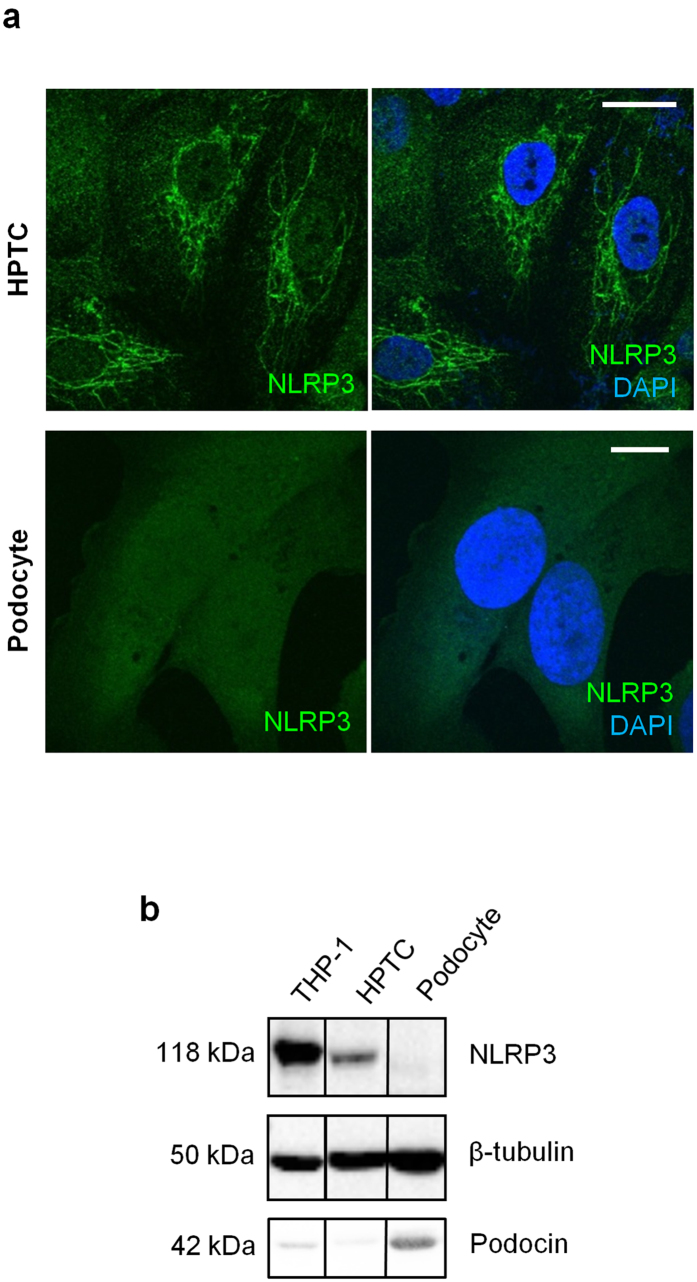
Subcellular localization of NLRP3 in HPTC and human podocyte cell cultures. (**a**) Indirect immunofluorescence in HPTC and immortalized, differentiated human podocytes probing for NLRP3 (green) in the presence of DAPI (blue) for merged images. Scale bar represent 20 μm (**b**) Immunoblotting for NLRP3, podocin and tubulin in lysates prepared from THP-1 cells (positive control), HPTC, or immortalized human podocytes. Representative of experiments performed at least 3 independent times.

**Figure 4:**
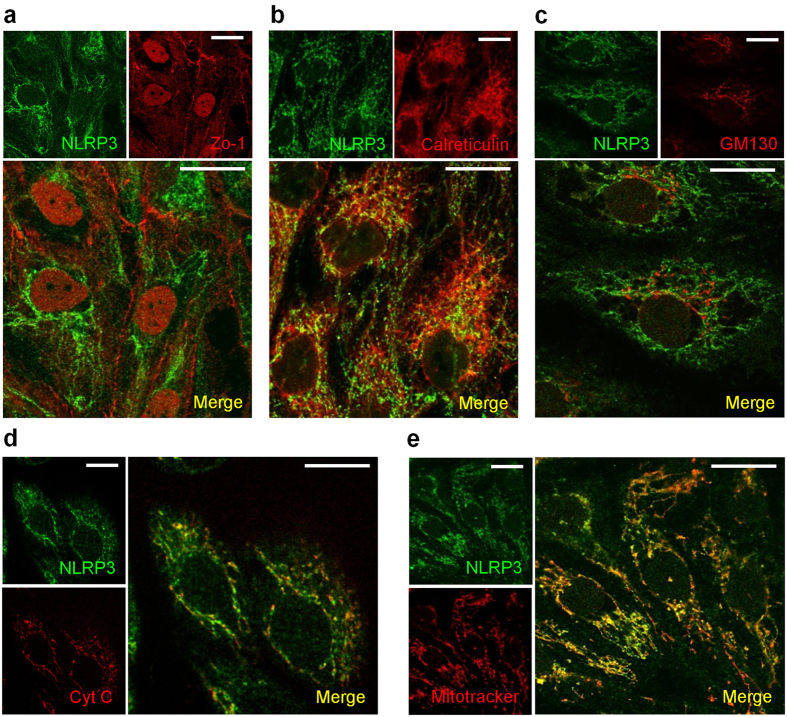
Subcellular localization of NLRP3 in HPTC. Indirect immunofluorescence probing for NLRP3 and (**a**) Zo-1, (**b**) calreticulin, (**c**) GM130, (**d**) cytochrome C, or (**e**) mitochondria (Mitotracker Red) in HPTC. Dual-labeling confocal microscopy shows colocalization of NLRP3 (green) with cytochrome C (red), Mitotracker Red and to a lesser extent calreticulin (red) in HPTC as shown in merged images (yellow denoting colocalization). Scale bar 20 μm. Images are representative of experiments performed at least 3 independent times.

Since NLRP3 is mainly expressed in tubules, *in vitro* studies were next performed to characterize intracellular NLRP3 expression and localization by immunocytochemistry. Consistent with our immunohistochemistry (IHC) data, NLRP3 expression was clearly detectable in primary human proximal tubular cells (HPTC) but not in a differentiated human podocyte cell line by immunofluorescence or immunoblotting (Fig. 3a,b). NLRP3 expression in HPTC with epithelial characteristics was confirmed by indirect IF microscopy co-staining for NLRP3 and the tight junction protein Zo-1 (Fig. 4a). At the cellular level, NLRP3 expression was confirmed in Zo-1 positive epithelial cells, but did not co-localize with Zo-1 *per se*. Isotype controls were negative (data not shown). NLRP3 has been proposed to localize to cytoplasm, mitochondrial-associated endoplasmic reticulum membranes (MAMs) and mitochondria in macrophages^7^,^25^,^26^ and to mitochondria in cardiac fibroblasts^27^. NLRP3 expression closely resembled the pattern of mitochondria in HPTC and significantly colocalized with mitochondrial-specific protein cytochrome C (Fig. 4d) and the mitochondrial dye, Mitotracker Red (Fig. 4e). NLRP3 also colocalized to a lesser extent with the MAMs marker calreticulin, but not to GM130, a Golgi complex protein (Fig. 4b,c). These data are consistent with prior studies and indicate that NLRP3 is a tubular epithelial expressed protein confined primarily to mitochondria and also to MAMs.

### NLRP3 mRNA and protein expression changes with prolonged TGF-β1 stimulation in HPTC

**Figure 5:**
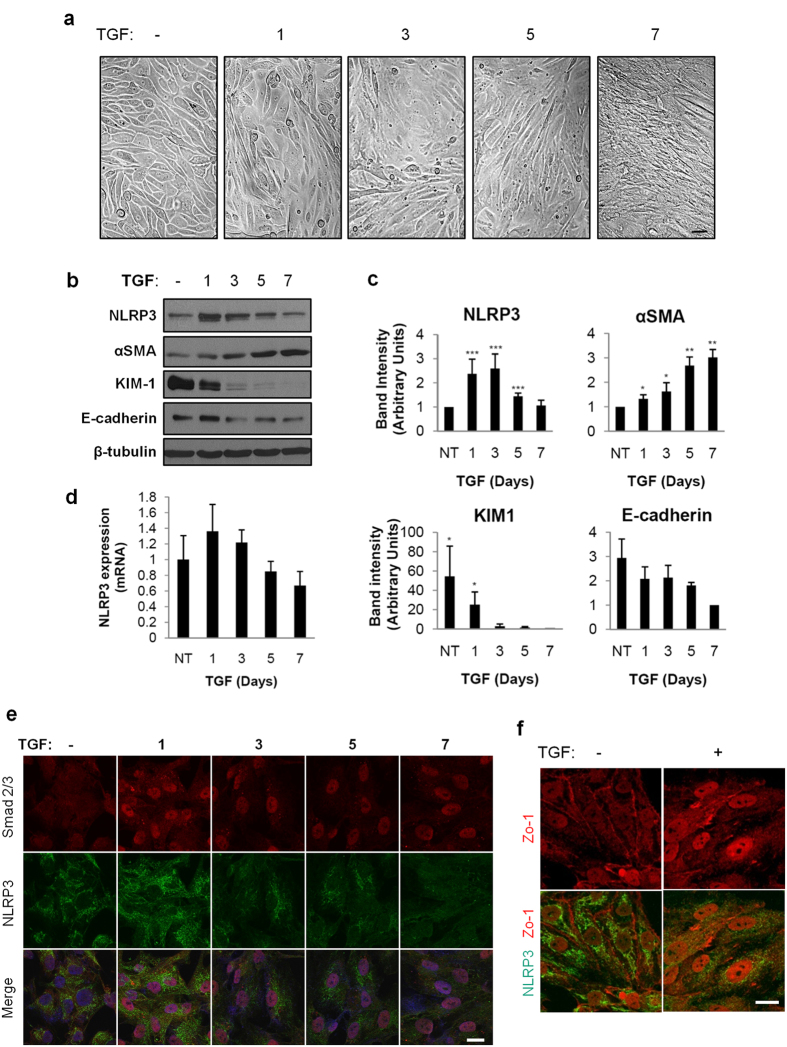
NLRP3 expression and subcellular localization in TGF-β1 stimulated HPTC. (**a**) Brightfield microscopy of HPTC treated with vehicle or TGF-β1 (5 ng/ml) for 1, 3, 5, or 7 days or vehicle control for 7 days. Scale bars represent 20 μm. (**b**) Lysates prepared from HPTC treated with 5 ng/ml TGF-β1 for 1, 3, 5 and 7 days to immunoblotting with the indicated antibodies. Cells treated with vehicle for 7 days were used as controls. Immunoblots are representative of at least four separate experiments. **(c**) Densitometric analysis of band intensity. Quantitation of bands by densitometry with mean densities obtained from at least four separate experiments (mean ± SD; *P < 0.05, **P < 0.01, ***P < 0.001). (**d**) NLRP3 mRNA expression by quantitative real-time PCR from RNA isolated from HPTC untreated or treated with 5 ng/ml TGF-β1 for 1, 3, 5 and 7 days or vehicle control for 7 days. mRNA expression levels were standardized to untreated samples. Values are normalized to endogenous 18S mRNA expression levels. Mean mRNA expression obtained from four separate experiments. (**e**) Indirect immunofluorescence probing for Smad 2/3 (red) and NLRP3 (green) in HPTC untreated or treated with TGF-β1 (5 ng/ml) for 1, 3, 5, and 7 days. Merged images show nuclear staining with DAPI. Scale bars represent 20 μm. (**f**) Indirect immunofluorescence probing for NLRP3 (green) and Zo-1 (red) in HPTC untreated or treated with TGF-β1 (5 ng/ml) for 7 days. Scale bars represent 20 μm. Images are representative of experiments performed 3 independent times.

**Figure 6:**
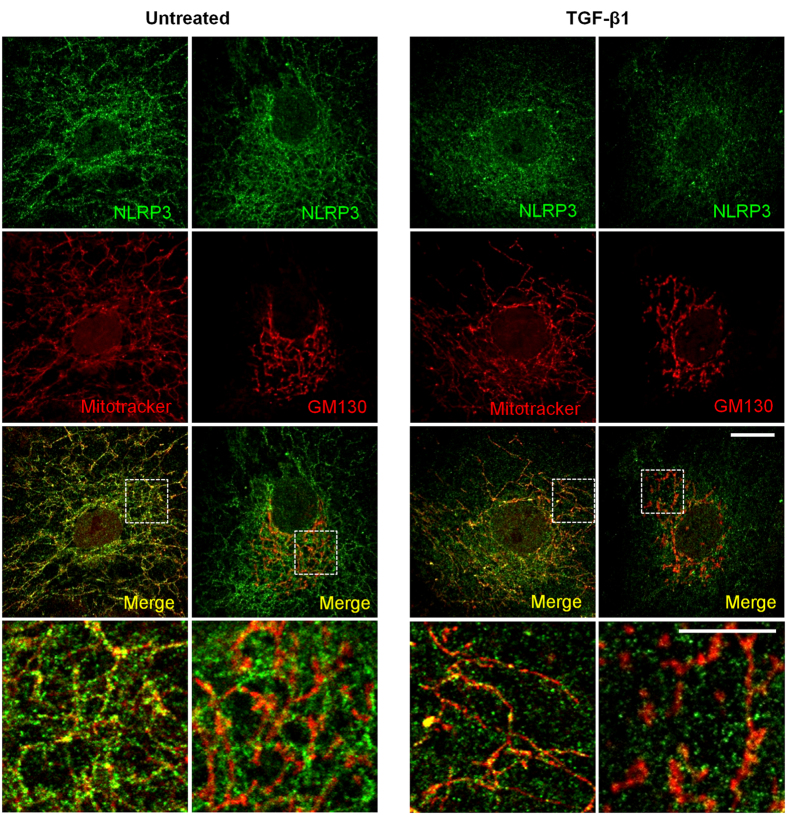
NLRP3 signal at mitochondria in TGF-β1 stimulated HPTC. Indirect immunofluorescence probing for NLRP3 (green) and GM130 (red) or mitochondria (Mitotracker Red) in HPTC untreated or treated with TGF-β1 (5 ng/ml) for 7 days. Bottom rows show magnified images of the outlined areas for single and merged images acquired from the red and green channels with yellow indicating colocalization. Scale bars represent 20 μm for full images and 10 μm for insets. Images are representative of experiments performed 3 independent times.

To further examine the biology of NLRP3 in the context of human tubular injury and fibrosis, we stimulated HPTC for up to seven days with TGF-β1 to model chronic epithelial cell injury and fibrosis *in vitro*. Following TGF-β1 stimulation, the HPTC gradually acquired a fibroblastic phenotype with an obvious progressive change in morphology most notable following seven days of treatment (Fig. 5a). Both NLRP3 protein (Fig. 5b,c) and mRNA (Fig. 5d) expression in HPTC peaked after treatment with TGF-β1 for three days. Beyond three days, the level of NLRP3 protein and mRNA expression decreased concomitantly with progression of TGF-β1 mediated phenotypic change that included increased α-SMA and reduced levels of E-cadherin (Fig. 5b–d). Consistent with the loss of epithelial cell phenotype induced by prolonged TGF-β1 stimulation in HPTC, the epithelial cell injury marker KIM-1 also decreased significantly over time (Fig. 5b,c). Following seven days of TGF-β1 stimulation, Smad 2/3 remained in the nucleus suggesting persistent activation of the Smad-dependent signaling pathway and clear loss of Zo-1 membrane staining suggesting loss of epithelial morphology (Fig. 5e,f). Interestingly, NLRP3 signal diminished at the mitochondria with preservation of the mitochondrial architecture suggesting reduced levels of NLRP3 at the mitochondria rather than structural compromise of the organelle itself (Fig. 6). Taken together, these data show that there is an initial rise in NLRP3 expression induced by TGF-β1, but ongoing stimulation and loss of epithelial phenotype in HPTC is associated with decreased NLRP3 protein and mRNA expression.

### NLRP3 regulation by ubiquitination

**Figure 7:**
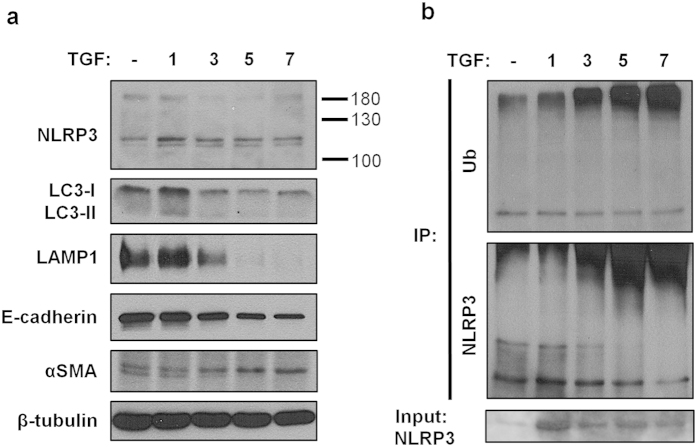
NLRP3 ubiquitination in TGF-β1 stimulated HPTC. (**a**) Lysates prepared from HPTC treated with 5 ng/ml TGF-β1 for 1, 3, 5 and 7 days were subjected to immunoblotting with the indicated antibodies. Cells treated with vehicle for 7 days was used as a control. (**b**) Immunoblotting of NLRP3 immunoprecipitates prepared from HPTC treated with TGF-β1 (5 ng/ml) for 1, 3, 5 and 7 days analyzed for ubiquitination. Untreated cells at 7 days was used as a control. Immunoblots are representative of 3 separate experiments.

Next we hypothesized that loss of NLRP3 signal at the mitochondria was a result of protein degradation mediated by either the ubiquitin-proteasome (UPS) or autophagy. The majority of intracellular proteins are degraded by the UPS pathway but aggregated proteins that are too large to enter the UPS pore are degraded by autophagy^28^,^29^. To examine the involvement of autophagy in NLRP3 degradation by TGF-β1, immunoblotting was performed probing for the autophagy activated protein LC3 (microtubule-associated protein light chain 3b). The pattern of LC3-I expression followed that of NLRP3 with the highest level at 24 hours of TGF-β1 treatment (Fig. 7a). Beyond 24 hours, there was no significant conversion to LC3-II, a marker for autophagosome formation. Furthermore, levels of LAMP-1 decreased indicating that the lysosomal machinery was not upregulated and was unlikely to play a role in NLRP3 degradation in response to TGF-β1 stimulation (Fig. 7a). Interestingly, high molecular weight bands that migrated above ~180 kDa (in addition to the usual mobility of NLRP3 predicted at 118 kDa) were consistently observed when probing for NLRP3 by immunoblotting suggesting a role for ubiquitination in NLRP3 protein turnover. To determine if TGF-β1 stimulation increased NLRP3 ubiquitination, NLRP3 was immunoprecipitated from control and TGF-β1 treated HPTC followed by immunoblotting for ubiquitin. Immunoprecipitated NLRP3 demonstrated a similar pattern of expression over 7 days of TGF-β1 stimulation as previously observed in total cellular lysates with NLRP3 levels highest at day one followed by a decreasing signal over time (Fig. 7b). Indeed, prolonged treatment with TGF-β1 resulted in a progressive increase in high molecular weight ubiquitinated NLRP3 signal that paralleled the progressive loss of NLRP3 signal at the expected mobility of ~118 kDa (Fig. 7b). These results show that under conditions of prolonged stimulation with TGF-β1, HPTC lose their epithelial phenotype and reduced NLRP3 protein expression in part by ubiquitin mediated degradation.

### Increased NLRP3 expression is associated with better clinical outcome in IgAN

**Figure 8:**
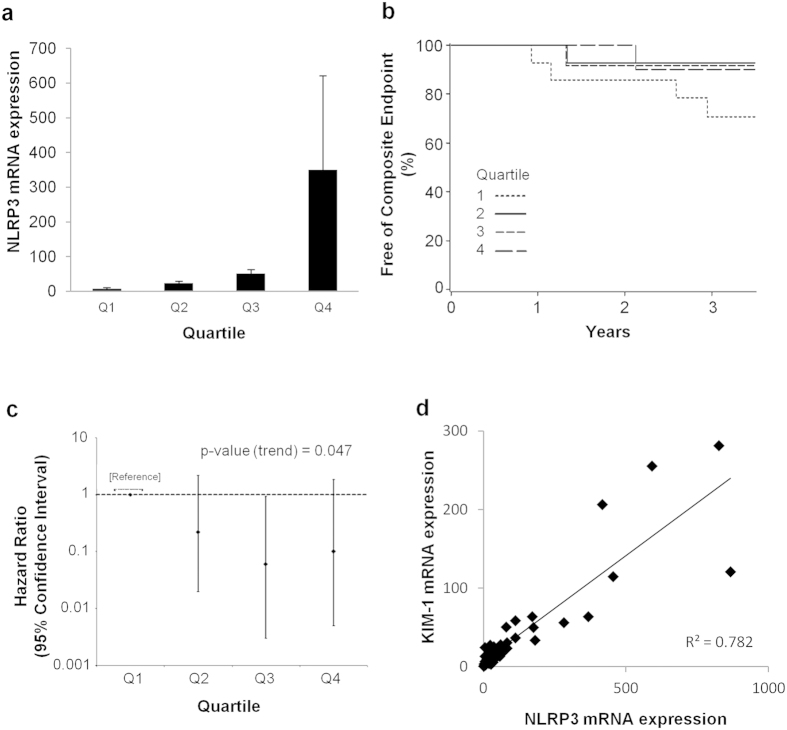
NLRP3 mRNA expression and renal outcome in IgAN. (**a**) NLRP3 mRNA expression by quantitative real-time PCR in patients with IgAN. RNA isolated from renal biopsies of patients with IgAN grouped into quartiles (Q1–4) based on NLRP3 mRNA expression levels standardized to normal kidney samples. Values are normalized to endogenous 18S mRNA expression levels. (**b**) Hazard ratio for NLRP3 quartiles based on the multivariable Cox model for the composite outcome of doubling serum Cr, ESRD or death. There is a significant linear trend towards lower risk of composite outcome with higher levels of NLRP3 (p = 0.047). (**c**) Product-limit survival estimates from each quartile. Quartile 1: low NLRP3; quartile 2: low-intermediate; quartile 3: high-intermediate; and quartile 4: high NLRP3. NLRP3 mRNA expression was analyzed as a logarithmic variable due to its non-normal distribution, and displayed as quartiles in a survival curve. (**d**) Correlation of NLRP3 mRNA expression with KIM-1 mRNA expression.

We previously demonstrated that NLRP3 mRNA expression in the kidney is increased in a limited number of patients with IgAN compared to normal^6^. To determine if NLRP3 expression correlated with IgAN disease outcome and progression, kidney NLRP3 mRNA expression was analyzed by real-time PCR in an expanded cohort of patients with IgAN and correlated to clinical outcomes. Fifty-four consecutive patients with a primary diagnosis of IgAN were selected for analysis from a pool of kidney biopsies performed between 2003 and 2007. The baseline characteristics of the cohort are shown in Table 1. Consistent with our previous study^6^ and the variable clinical presentation of IgAN, NLRP3 mRNA expression in kidney biopsies varied from two to several hundred-fold above normal controls (Fig. 8a). NLRP3 mRNA expression levels in IgAN patients were arbitrarily categorized into quartiles and correlated to the time to event of the composite endpoint of doubling of serum creatinine, ESRD or death. Interestingly, there was a trend towards for a lower risk of the composite endpoint in IgAN patients with high expression of NLRP3 (Fig. 8b). In a multivariable Cox model for the composite outcome of doubling of serum creatinine, ESRD or death adjusted for histopathological features according to the Oxford classification, there was a significant linear trend (p-value 0.047) towards lower risk of the composite outcome among patients with higher quartiles of NLRP3 (Fig. 8c). Similar to results reported by other groups^30^,^31^, the multivariable Cox model also demonstrated a significantly increased risk of the composite outcome associated with a tubular atrophy/interstitial fibrosis score of T = 2 by the Oxford classification (p-value 0.01, Table 2). Given that NLRP3 is localized primarily to the tubular compartment, these observations suggest that NLRP3 expression in the kidney decreases during tubular atrophy/interstitial fibrosis observed in progressive IgAN that is associated with a worse clinical outcome. Consistent with this premise, NLRP3 mRNA expression in IgAN directly correlated with KIM-1 (also a tubular activation marker) with an R-squared value of 0.782 (Fig. 8d).

## Discussion

In this study, we have demonstrated for the first time a relationship between NLRP3 expression and human disease progression in IgAN. Using a combination of *in vitro* studies in human proximal tubular cells with clinical correlation to human kidney biopsy samples from patients with IgAN, we have established that NLRP3 is primarily expressed in the kidney tubular epithelium in humans with reduced expression at the transcription and post-translational level during tubular injury *in vitro* and *in vivo*. These data suggest that NLRP3 represents a marker of tubular activation and tubular integrity that is progressively lost during chronic renal injury, tubular atrophy and fibrosis. Together, our data provide a perspective on the biology of NLRP3 in the context of human kidney disease.

To better characterize NLRP3 in human kidney disease, our study carefully analyzed NLRP3 localization in kidney tissue using various antibodies and fixative methods. The localization of NLRP3 in kidney tissue has been inconsistent with studies emphasizing the role for NLRP3 in podocytes^10^,^20^. In several prior studies, NLRP3 can clearly be seen at tubules in human tissue but the relevance of NLRP3 tubular localization has never been addressed by the authors^21^,^32^. Furthermore, a recent study by Liu’s group demonstrated NLRP3 expression at tubules in albumin overloaded rats^33^. We found that NLRP3 predominantly localized to the tubules with virtually no staining in glomeruli in histologically normal kidney. In IgAN, there was detectable NLRP3 at the glomeruli but NLRP3 expression remained higher in tubules. Thus we focused on the tubular expression of NLRP3 since we previously demonstrated TGF-β1 regulation of NLRP3 in HPTC^7^ and tubular atrophy/interstitial fibrosis (rather than mesangial hypercellularity, endocapillary hypercellularity or segmental glomerulosclerosis) remains the best predictor of outcome in IgAN based on the Oxford classification^31^. The immunostaining results and mRNA expression in the human kidney corroborate the high levels of NLRP3 expression (protein and mRNA) seen in primary HPTC cultures. Conversely, the loss of NLRP3 expression during epithelial injury *in vitro* are consistent with reduced NLRP3 expression in patients with significant tubulointerstitial disease and progressive IgAN.

One of the primary goals of this study was to translate NLRP3 knowledge to human kidney disease. In the NLR family of genes, humans contain one copy of each of the 22 NLR genes that includes NLRP3. In mice, several NLR genes (e.g. NLRP1, NLRP9 or NAIP) are very pleomorphic and certain genes (e.g. NLRP7, NLRP8, NLRP11) are not present^34^. Based on experimental models in mice, NLRP3 is suggested to contribute to kidney injury, inflammation and fibrosis; however, these paradigms have not yet been applied to human disease. This study is the first to extensively characterize NLRP3 in the context of human kidney disease using only human biopsy tissues and primary, low passage human cells. IgAN was used as a disease focus in this study since it is a chronic, progressive kidney disease with pathology in both glomerular and tubulointerstitial compartments and features of chronic inflammation, tubular injury and fibrosis where NLRP3, based on experimental data, is suggested to play a role^6^,^7^. Our results suggest that lower levels of NLRP3 mRNA expression are related to a worse prognosis in IgAN patients. Though surprising at first, our data has identified NLRP3 as a predominantly tubular protein in the kidney that decreases at the mRNA and protein levels in association with loss of tubular epithelial phenotype and cellular demise. Thus, in the context of progressive tubular atrophy and increased renal fibrosis, a loss of NLRP3 expression in advancing IgAN becomes plausible. The strong correlation between NLRP3 expression and the tubule-restricted marker KIM-1 supports this premise.

The data contained in this study are unable to inform a functional role for NLRP3 in IgAN pathogenesis. The activation of NLRP3 is a multistep process that first requires signals to upregulate gene/protein expression followed by a second signal to activate NLRP3 function or the inflammasome^35^. Our data and prior studies demonstrate that signaling downstream of the TGF receptor and Toll-like receptors increases NLRP3 expression in HPTC^6^,^7^. Given the significant alteration in renal gene expression observed in IgAN that includes TGFβ, it is likely that multiple pathways contribute to the upregulation of NLRP3 observed in our patient biopsies^36^,^37^,^38^,^39^. However, the potential function of NLRP3 in IgAN remains to be determined. While both canonical (inflammasome-dependent) and non-canonical (inflammasome-independent) roles for NLRP3 have been demonstrated in the glomerulus and tubular epithelium in experimental kidney disease^5^,^7^,^20^,^21^, increased NLRP3 gene or protein expression in IgAN does not imply function given the requirement for a second activating signal. Thus, the data are hypothesis-generating and provide the basis for further studies of NLR biology in human kidney disease. To address NLRP3 function in human kidney disease will require the development of gene targeting strategies in low passage, primary cell cultures, specific pharmacologic therapies and ongoing characterization of biomarkers downstream of canonical and non-canonical NLRP3 pathways. Currently, these approaches remain technically challenging, but the focus of future research.

A direction towards molecular analysis of IgAN is warranted since studies to date using clinical and histopathologic parameters have yielded limited capabilities to predict disease progression. During the development of the Oxford classification of IgAN, tubular atrophy/interstitial fibrosis had the highest odds ratio for risk of progression^31^. More recently, mesangial hypercellularity was also reported to be a significant factor for IgAN progression^30^. Thus, the validation of tubular biomarkers in IgAN may prove to be a useful adjunct to clinicopathologic scoring systems to identify patients with preserved renal mass and/or treatment responsive disease. Our data suggest that NLRP3 may potentially be developed as a biomarker for tubular integrity and disease chronicity in IgAN but is limited by the retrospective nature of the clinical data, a relatively small sample size, and the reliance on de-identified archived laboratory and biopsy data. Although the analysis included the major biopsy features (MEST score), eGFR and proteinuria that remain the strongest prognostic factors identified in IgAN^40^, a more thorough evaluation that included co-morbid and treatment data may have provided further insight and strengthened the study conclusions. A future prospective study to develop and validate NLRP3 as a biomarker in IgAN would be needed that includes a larger cohort, expanded clinical parameters including treatment data and potentially a urine-based assay before any practical application can be developed regarding NLRP3 in IgAN. Furthermore, given the vast differences in pathogenesis, whether or not NLRP3 represents a similar biomarker in other forms of kidney disease cannot be determined from these results.

Our study is the first to extensively characterize NLRP3 in the human kidney and evaluate its expression in the context of IgAN, a common cause of chronic kidney disease. Furthermore, the analysis of NLRP3 using a purely human system *in vitro* and *in vivo* is the first step in the bench to the bedside knowledge translation of NLRs in human kidney disease. Our findings provide important perspective on the interpretation of animal and other *in vitro* studies that examine the role of NLRP3 in experimental renal injury and provide a basis for future studies of NLRs in the human kidney.

## Materials and Methods

### Primary tubular epithelial cell culture and immortalized human podocyte cell line

Human proximal tubular epithelial cells (HPTC) were prepared as previously described^6^,^41^. Cells were derived from disease-free tissue margins of total or partial nephrectomies (slated for disposal) obtained from patients undergoing medically indicated surgery for renal tumors. All patients had normal kidney function and no known chronic kidney disease prior to nephrectomy. All data/samples were completely de-identified by the custodians of the materials and thus patient-identifying information was not available to investigators in this study. Human nephrectomy sample collection was approved and conducted in accordance with guidelines set forth by the Conjoint Health Research Ethics Board at the University of Calgary and Alberta Health Services. Kidney tissues were collected within 2 hours of surgery in HBSS supplemented with 5 mg/ml of penicillin/streptomycin on ice. Samples were inspected and confirmed to have clear margins and be devoid of gross disease. A portion of kidney tissue was removed for PCR, immunoperoxidase and immunofluorescence studies as described below. Following removal of the renal capsule, remaining cortical tissue was cut and minced. After digestion in collagenase (1.5 mg/ml in HBSS) at 37 °C for 60 minutes, samples were passed through serial filters from 200 μm to 45 μm to remove intact glomeruli and large cellular debris. Samples were cultured on uncoated plates at 37 °C for 90 minutes in K1 culture medium (DMEM/F12 containing 10% FBS, 1% penicillin-streptomycin, 125 ng/ml prostaglandin E1, 10 ng/ml EGF, 1.8 μg/ml L-thyroxine, 3.38 ng/ml hydrocortisone, and 2.5 mg/ml insulin-transferrin-sodium selenite supplement, all reagents from Sigma-Aldrich). HPTC were collected and cultured onto collagen IV coated cell culture plates. To maintain the epithelial phenotype, HPTC were used less than 2 passages. Prior to stimulation with TGF-β1 (5 ng/ml), cells were passaged onto collagen coated plates or glass coverslips and grown overnight to the desired confluency. Experiments using conditionally immortalized human podocyte cell line were performed in the laboratory of Dr. Martin Pollak (Beth Israel Deaconess Medical Center/Harvard Medical School). Cells were cultured in basic growth medium as previously described and differentiated for 14 days prior to fixation or preparation of lysates^42^.

### Antibodies

Antibodies were from the following sources: anti-mouse α-smooth muscle actin (Clone 1A4; Sigma-Aldrich), mouse anti-human β-tubulin (Sigma), rabbit anti-human NLRP3 (Sigma), mouse anti-human NLRP3 (Cryo-2, Adipogen), rabbit anti-human Smad 2/3 (Santa Cruz Biotechnology), mouse anti-human KIM-1/TIM-1 (R&D Systems), mouse anti-human E-cadherin (clone 36; BD Biosciences), rabbit anti-human Zo1 (Invitrogen), rabbit anti-human calreticulin (Abcam), rabbit anti-human cytochrome C (Abcam), rabbit anti-human GM130 (Abcam), and rabbit anti-human podocin (Sigma). All primary antibodies were used at a dilution of 1:1000 for immunoblotting and 1:200 for immunofluorescence respectively unless otherwise specified. For mitochondrial labelling, 200 nM of Mitotracker Red CMXRos (Invitrogen) was added to the culture medium and cells incubated for 15 min prior to fixation of cells.

### Immunohistochemistry

Human kidney tissues frozen in optimal cutting temperature (OCT) media or embedded in paraffin were used in this study. Paraffin-embedded samples were deparaffinized using xylene and ethanol washes. Antigen retrieval was performed using citric acid buffer (10 mM citric acid pH 6.0) or EDTA Buffer (1 mM EDTA, 0.05% Tween 20, 10 mM Tris, pH 9.0) in a steamer for 30 min. Sections were blocked for 30 min in IHC (immunohistochemistry) blocking solution (1% BSA, 2% goat serum, 0.1% Triton X-100 in PBS). For immunoperoxidase staining, sample endogenous peroxidase activity was quenched with 0.5% hydrogen peroxide. Samples were then incubated with primary antibodies prepared in 1:50 dilutions of blocking solution for 2 hours followed by PBS washes and incubation with 1:500 dilutions of mouse or rabbit HRP-conjugated secondary antibody. EnVision™+ Dual Link System-HRP (Dako) was used for DAB staining as per manufacturer’s instructions. Counterstaining and dehydration was performed using Harris hematoxylin solution (Sigma-Aldrich) and sequential washes in graded ethanol and xylene. Cells were mounted with ProLong® Gold antifade reagent in the presence of DAPI (Invitrogen).

### Immunofluorescence of cell culture and kidney tissue

HPTC grown on glass coverslips were fixed with 4% paraformaldehyde, quenched with 50 mM ammonium chloride and permeabilized with 0.1% Triton X-100 + 0.05% SDS. Cells were blocked with 0.2% gelatin in PBS. Podocytes were blocked with 3% BSA in PBS. Cells were incubated with primary antibody for 60 min followed by incubation with Alexa Fluor 488 or 568 labelled secondary antibodies (Invitrogen) for 60 min. Cells were mounted with ProLong® Gold antifade reagent in the presence of DAPI (Invitrogen). Confocal images were acquired using a FV1000 laser scanning microscope (Olympus) with a 60X oil lens (NA 1.4). NLRP3 and PDGFR-β IF was performed by using 5 μm frozen kidney sections. IF of tissue samples was performed essentially as described for cell culture except using IHC blocking solution (1% BSA, 2% goat serum, 0.1% Triton X-100 in PBS) for 30 min. There was an additional step of incubating tissue samples in 0.1% Sudan black B (Sigma; in 70% ethanol) prior to mounting with ProLong® Gold antifade reagent.

### Immunoblotting

Cell lysates were separated on SDS-PAGE and then transferred to nitrocellulose membranes. Membranes were blocked with 5% skim milk proteins in PBS/0.05%Tween and then incubated with the appropriate primary antibodies overnight at 4 °C. Immunoblots were washed, incubated with the appropriate secondary horseradish peroxidase-conjugated antibodies, and visualized with ECL chemiluminescence (GE Healthcare). ImageJ (version 1.47) was used quantify band intensity by densitometry. At least four experiments were used to determine the averages and standard deviations for each treatment.

### Immunoprecipitation

NLRP3 ubiquitination was detected in control and TGF-β1 stimulated cells lysed in 1% NP-40 buffer (50 mM Tris-HCl [pH 7.4], 150 mM NaCl, 5 mM EDTA, 1% NP-40, 1 mM PMSF, 1 mM Na_3_VO_4_, Roche protease inhibitors) or RIPA buffer (50 mM Tris-HCl [pH 8.0], 150 mM NaCl, 1 mM EDTA, 0.05% sodium deoxycholate, 1% Triton X-100, 0.1% SDS, 1 mM PMSF, Roche protease inhibitors). NLRP3 was immunoprecipitated overnight at 4 °C using 0.01 μg/μl of anti-NLRP3 antibody (Cryo-2, Adipogen) and 10 μl of packed protein G sepharose beads (GE Healthcare) per 100 μl of lysate. Immunoprecipitated complexes were then washed extensively in lysis buffer and proteins were separated by SDS-PAGE and analyzed by immunoblotting.

### Human kidney sample selection

All consecutive biopsies with a primary diagnosis of IgA nephropathy were selected from September 2003 to December 2007 for analysis of NLRP3 mRNA expression by quantitative real-time PCR. Biopsies of poor quality or lacking glomeruli were excluded from the analysis. Patients with advanced renal failure (GFR ≤ 15 ml/min/1.73 m^2^) were also excluded to avoid patients with stage 5 CKD. Fifty-four patient biospies were available for this study. All biopsy samples were secondary use of archived material previously collected for clinical care. Histologically normal kidney tissue from nephrectomy samples as collected above were used as controls (n = 10). All data/samples were completely de-identified by the custodians of the materials and thus patient-identifying information was not available to investigators of this study. The study protocol was approved and conducted in accordance with the guidelines set by the Conjoint Health Research Ethics Board at the University of Calgary and Alberta Health Services.

### Real-time PCR

Human NLRP3 mRNA expression was prepared using total RNA extracted from frozen human kidney biopsy samples obtained from the Biobank for the Molecular Classification of Kidney Disease at the University of Calgary and Calgary Laboratory Services. Tissue samples were homogenized and total RNA from tissue was isolated using QIAshredder and an RNeasy Mini kit (Qiagen) as per manufacturer’s instruction. For human kidney samples, reverse transcription was carried out using 10–50 ng RNA, random hexamers (3 μg/μl), M-MLV reverse transcriptase (200 U/μl, Invitrogen) as per manufacturer’s protocol. The sequences for human NLRP3 primers and probe were as follows: forward primer 5′- TGAAGAAAGATTACCGTAAGAAGTACAGA-3′, reverse primer 5′-GCGTTTGTTGAGGCTCACACT-3′, and probe 6-FAM-5′-AATGCCCGTCTGGGTG-3′-MGB (Applied Biosystems). The 20X 18S rRNA FAM/MGB Probe (Applied Biosystems) was used as the endogenous control. Target gene reactions were formed using 10 μl 2X Taqman Universal PCR Master Mix (Applied Biosystems), 900 nM of each primer, 200 nM probe and 2.5 μl cDNA template (diluted 1:5 from original). Amplification was performed in 96-well reaction plates using the 7900HT Fast Real-Time PCR System (Applied Biosystems) and results analyzed with SDS 2.3 and RQ Manager software. Four experiments were used to determine the averages and standard deviations for each treatment.

### Pathology review using the Oxford classification for IgAN

Pathology scoring for tubular injury was performed in a blinded manner on hematoxylin/eosin, periodic acid-Schiff, Schiff-methenamine silver, Congo red and Masson trichrome stained slides. Renal biopsies were scored according to the Oxford Classification: mesangial hypercellularity, M0/M1; endocapillary hypercellularity, E0/E1; segmental glomerulosclerosis, S0/S1; tubular atrophy/interstitial fibrosis, T0/T1/T2 (<25%/25–50%/>50%). Biopsy samples had a minimum of eight glomeruli available for examination by light microscopy.

### Statistical analysis

All cell culture experiments were performed at least in triplicates. Statistical analysis was performed using Microsoft Excel software and Statistical Analysis Software (SAS) version 9.3 with a type one error rate of 0.05 considered significant. All patients were de-identified for the study, and data collected using a unique patient health number. For time to event analyses all available outpatient serum creatinine measures in the province of Alberta were used to identify doubling of serum creatinine, using the closest outpatient creatinine value prior to biopsy as the baseline measurement. ESRD and death were identified from the records of the Southern Alberta Renal Program^43^. Subjects were followed from the time of their index kidney biopsy to March 31, 2009 or the time of the first occurrence of death, ESRD requiring dialysis, or doubling of serum creatinine. NLRP3 gene expression in 54 patients was divided into quartiles due to its non-normal distribution. Time to the composite outcome of death, ESRD or doubling of serum creatinine was plotted using the Kaplan-Meier method stratified by NLRP3 quartiles. A Cox proportional hazards model was fit for the composite outcome including independent variables for each of the Oxford classification variables and quartiles of NLRP3 expression. The proportional hazards assumption was tested and met. For analysis of *in vitro* data, GraphPad Prism 5 was used to calculate unpaired student t-tests.

## Additional Information

**How to cite this article**: Chun, J. *et al.* NLRP3 Localizes to the Tubular Epithelium in Human Kidney and Correlates With Outcome in IgA Nephropathy. *Sci. Rep.* **6**, 24667; doi: 10.1038/srep24667 (2016).

## Supplementary Material

Supplementary Information

## Figures and Tables

**Table 1:** Baseline characteristics of the study population with IgAN.

Characteristic	Quartile 1	Quartile 2	Quartile 3	Quartile 4	Overall
Sample size	14	14	13	13	54
Age, years (SD)	45.2 (17.3)	43.1 (13.4)	43.6 (11.6)	41.2 (15.6)	43.3 (14.3)
Sex (% Male)	9 (64.3%)	8 (57.1%)	8 (61.5%)	9 (69.2%)	34 (63.0%)
Proteinuria*					
Heavy	5 (35.7%)	6 (42.9%)	5 (38.5%)	7 (53.9%)	23 (42.6%)
Mild	5 (35.7%)	3 (21.4%)	1 (7.7%)	2 (15.4%)	11 (20.4%)
Normal	2 (14.3%)	1 (7.1%)	3 (23.1%)	0 (0%)	6 (11.1%)
Not measured	2 (14.3%)	4 (28.6%)	4 (30.8%)	4 (30.8%)	14 (26.0%)
Follow up years (SD)	2.9 (1.1)	2.6 (1.4)	3.0 (1.2)	2.9 (1.2)	2.9 (1.2)
Oxford classification					
M	1 (7.1%)	3 (21.4%)	3 (23.1%)	3 (23.1%)	10 (18.5%)
E	9 (64.3%)	11 (78.6%)	11 (84.6%)	7 (53.9%)	38 (70.4%)
S	12 (85.7%)	11 (78.6%)	12 (92.3%)	10 (76.9%)	45 (83.3%)
T1	4 (28.6%)	6 (42.9%)	4 (30.8%)	4 (30.8%)	18 (33.3%)
T2	1 (7.1%)	0 (0%)	3 (23.1%)	1 (7.7%)	5 (9.26%)
Baseline eGFR (SD)	70.9 (37.1)	82.1 (24.7)	54.1 (21.3)	67.2 (32.6)	69.2 (30.6)
NLRP3 mRNA expression range	0.9–14.3	14.5–32.9	35.5–63.7	79.2–866.8	0.9–866.8

54 patients with IgAN separated into quartiles for age, degree of proteinuria. Patients with advanced renal failure (eGFR ≤ 15 ml/min) were excluded from the analysis to avoid patients with ESRD. Abbreviations: eGFR, estimated glomerular filtration rate (ml/min/1.73 m^2^); Oxford classification for IgAN: M, mesangial hypercellularity; E, endocapillary hypercellularity; S, segmental glomerulosclerosis; T, tubular atrophy/interstitial fibrosis; T1 = 26–50%, T2 = >50%. SD, standard deviation. *Proteinuria categorized based on urine microalbumin to creatinine ratios (ACR) or urinalysis (UA) if ACR unavailable. Normal = ACR < 3 mg/mmol or 0; Mild = 3–30 mg/mmol or trace/1+; Heavy = >30 mg/mmol or ≥2+.

**Table 2:** Multivariable Cox model for the composite outcome of doubling of serum creatinine, ESRD, or death correlated to the pathologic features of the Oxford classification.

Predictors	Hazard ratio (95% CI)	p-value
Mesangial hypercellularity	5.90 (0.78, 44.44)	0.085
Endocapillary hypercellularity	1.29 (0.11, 15.26)	0.839
Segmental glomerulosclerosis	ND (0, –)	0.995
Tubular atrophy/interstitial fibrosis T1 (vs. T0)	1.73 (0.16, 18.59)	0.652
T2 (vs. T0)	43.82 (2.61, 734.44)	0.01*

T = tubular atrophy/interstitial fibrosis. T0 = 0–25%, T1 = 26–50%, T2 = >50%.

*Indicates statistically significance p < 0.05. ND, not determined. Due to limitations of modeling, the hazard ratio for segmental sclerosis could not be determined since all biopsied samples had the presence of segmental glomerulosclerosis.
