# Supplementary material for: NLRP3 Localizes to the Tubular Epithelium in Human Kidney and Correlates With Outcome in IgA Nephropathy
Source: Sci Rep. 2016 Apr 20;6:24667. doi: 10.1038/srep24667 (PMC4837396; doi:10.1038/srep24667)
Supplement: Supplementary Information [file srep24667-s1.pdf]

NLRP3 Localizes to the Tubular Epithelium in Human Kidney and Correlates With Outcome in IgA Nephropathy

Justin Chun[*], Hyunjae Chung[*], Xiangyu Wang[*], Rebecca Barry[*], Zohreh Mohammad Taheri[‡], Jaye M. Platnich*, Sofia B. Ahmed[*], Kiril Trpkov[‡], Brenda Hemmelgarn[*], Hallgrimur Benediktsson[‡], Matthew James[*], and Daniel A Muruve[*,∥]

[*]Department of Medicine, [‡]Department of Pathology and Laboratory Medicine, Snyder Institute for Chronic Diseases, University of Calgary, Calgary, Alberta, Canada.

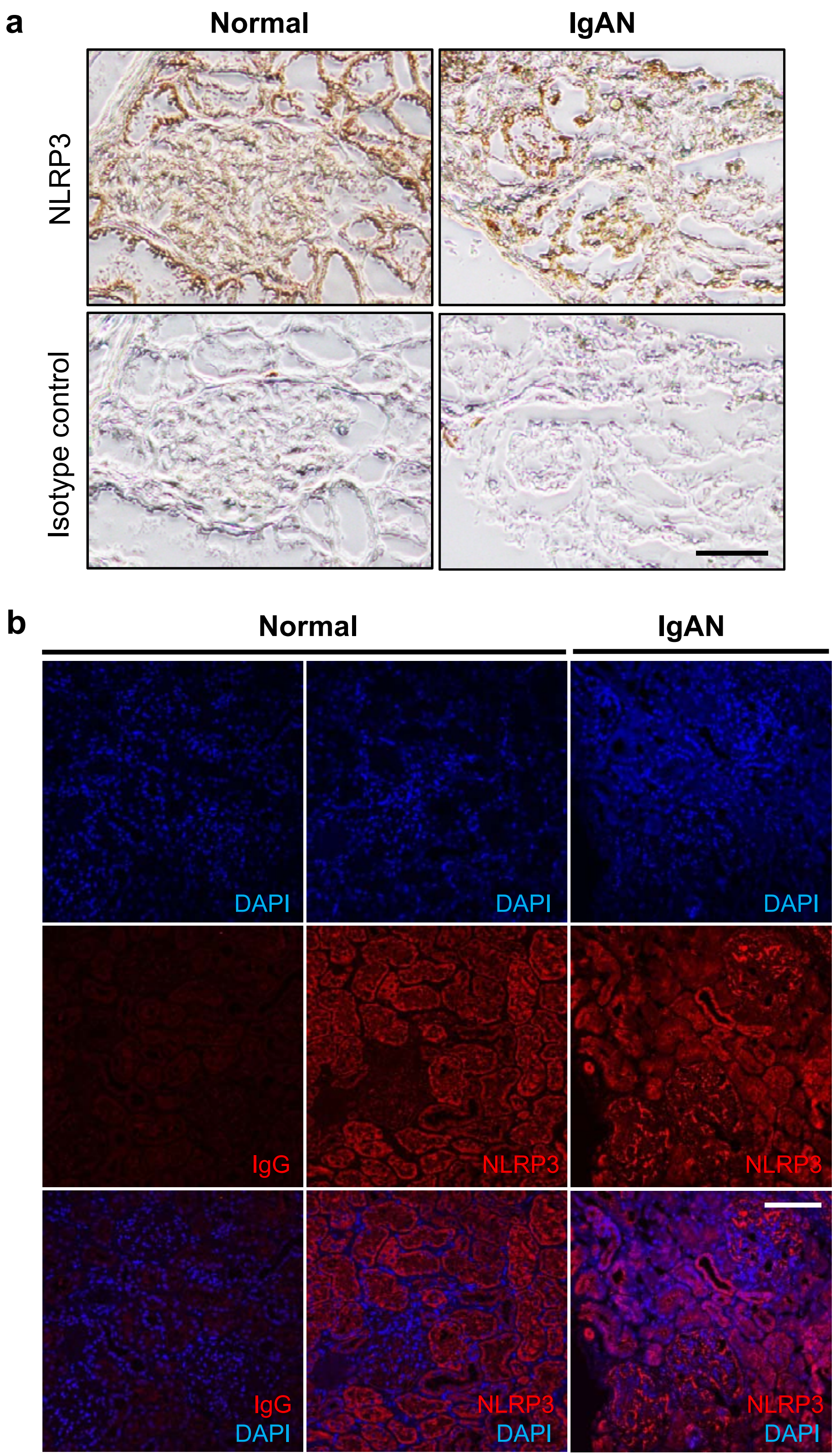

Supplementary Figure S1. Mouse IgG isotype control and NLRP3 expression in human kidney tissue. (**a**) Sections of frozen tissue from representative nephrectomy sample with normal margins or biopsy from a patient with IgAN were immunoperoxidase stained with mouse monoclonal NLRP3 (Cryo2) or mouse IgG isotype control antibodies without counterstaining with hematoxylin. Scale bar represents 100 μm. (**b**) Indirect immunofluorescence for NLRP3 (Cryo2; red) or mouse IgG (red) with DAPI (blue) in paraffin embedded tissue from a nephrectomy with normal margins or a biopsy from a representative patient with IgAN. Scale bar represents 100 μm.
